# Peripheral blood gene expression profiles in COPD subjects

**DOI:** 10.1186/2043-9113-1-12

**Published:** 2011-04-24

**Authors:** Soumyaroop Bhattacharya, Shivraj Tyagi, Sorachai Srisuma, Dawn L DeMeo, Steven D Shapiro, Raphael Bueno, Edwin K Silverman, John J Reilly, Thomas J Mariani

**Affiliations:** 1 Neonatology Division and Center for Pediatric Biomedical Research, University of Rochester Medical Center, 601 Elmwood Avenue, Rochester, 14642, NY; 2 Pulmonary and Critical Care Division, Department of Medicine, The Channing Laboratory, Brigham and Women's Hospital, Harvard Medical School, 181 Longwood avenue, Boston, 02115, MA; 3 Thoracic Surgery, Brigham and Women's Hospital, Harvard Medical School, 15 Francis Street, Boston, 02115, MA; 4 Department of Physiology, Faculty of Medicine Siriraj Hospital, Mahidol University, 2 Prannok Road, Bangkok Noi, Bangkok, 10700, THAILAND; 5 Department of Medicine, University of Pittsburgh Medical Center, 3550 Terrace StreetPittsburgh, 15261, PA

**Keywords:** Microarray, Biomarkers, PBMC

## Abstract

To identify non-invasive gene expression markers for chronic obstructive pulmonary disease (COPD), we performed genome-wide expression profiling of peripheral blood samples from 12 subjects with significant airflow obstruction and an equal number of non-obstructed controls. RNA was isolated from Peripheral Blood Mononuclear Cells (PBMCs) and gene expression was assessed using Affymetrix U133 Plus 2.0 arrays.

Tests for gene expression changes that discriminate between COPD cases (FEV_1_< 70% predicted, FEV_1_/FVC < 0.7) and controls (FEV_1_> 80% predicted, FEV_1_/FVC > 0.7) were performed using Significance Analysis of Microarrays (SAM) and Bayesian Analysis of Differential Gene Expression (BADGE). Using either test at high stringency (SAM median FDR = 0 or BADGE p < 0.01) we identified differential expression for 45 known genes. Correlation of gene expression with lung function measurements (FEV_1 _& FEV_1_/FVC), using both Pearson and Spearman correlation coefficients (p < 0.05), identified a set of 86 genes. A total of 16 markers showed evidence of significant correlation (p < 0.05) with quantitative traits and differential expression between cases and controls. We further compared our peripheral gene expression markers with those we previously identified from lung tissue of the same cohort. Two genes, RP9and NAPE-PLD, were identified as decreased in COPD cases compared to controls in both lung tissue and blood. These results contribute to our understanding of gene expression changes in the peripheral blood of patients with COPD and may provide insight into potential mechanisms involved in the disease.

## Introduction

Chronic obstructive pulmonary disease (COPD), an inflammatory disorder that is characterized by a slowly progressive development of irreversible airflow limitation, is currently the fourth leading cause of death in the United States. Sixteen million Americans live with the disease, and there are 800 million affected individuals worldwide. Strongly associated with cigarette smoking, COPD is expected to be the third most common cause of death and fifth most common cause of disability worldwide by 2020[1]. COPD is typically diagnosed late in life, and late in the course of disease when the patient presents with significant physiological impairment [2,3]. The need for improved early diagnosis and the identification of novel therapeutic targets for this debilitating disease has recently gained heightened interest.

Chronic obstructive bronchitis/bronchiolitis with peribronchiolar fibrosis (small airways disease), and abnormal enlargement of airspace distal to the terminal bronchioles with destruction of lung parenchyma (emphysema) are the pathological hallmarks of disease. Small airways disease and emphysema can present alone or in combination, with varying degrees of severity [4,5]. COPD is now considered primarily an inflammatory disorder involving abnormalities in both innate and adaptive immune responses. Inflammatory abnormalities in COPD include a significant increase in macrophage numbers in the lung and alveolar space, at the sites of alveolar destruction. Increased macrophage numbers may be due to increased monocyte recruitment and may result in higher secretion of inflammatory proteins leading to pathophysiological features of COPD [6]. However, systemic impairments have also been observed in patients [7].

Environmental factors contribute to varying susceptibility to COPD in the general population with the greatest environmental exposure in developed countries being tobacco smoke[8,9]. Exposure to other airborne pollutants, such as ozone, also appears to increase risk. While an increasing rate of smoking contributes to the growing incidence of COPD in developing countries as well, indoor air pollution associated with heating and cooking fuel is the major environmental risk factor, contributing to almost 3% of the global burden of disease [10]. In addition to environmental risk factors, varying genetic susceptibility to COPD exists among individuals, particularly with respect to the response to cigarette smoke [11,12]. Given the complexity of disease pathogenesis, the presence of varying levels of susceptibility in the general population and the fact that patients rarely present early in disease pathogenesis (at a time when disease-modifying therapy may be more effective) the identification of biological markers of disease susceptibility and/or progression are needed.

Numerous previous studies have sought to identify disease biomarkers in various forms, such as genetic or expression variants. DNA microarrays have been proven to be a powerful tool capable of biomarker discovery for various disease states. Multiple groups have previously applied microarray analysis to identify gene expression changes associated with COPD [13-16]. All these studies have used lung tissues obtained through invasive surgical procedures. Application of discovery approaches to samples derived from minimally-invasive procedures may provide biomarkers for diagnosis and therapeutic management of COPD. One previous study used whole blood to search for novel protein markers of COPD[16]. Here, we present a novel gene expression microarray data set generated from PBMC isolated from 24 subjects with varying levels of airflow obstruction.

## Methods

### Sample Collection

This study was approved by the Partners Health Care Human Research Committee. Peripheral blood, along with lung tissue, was obtained from 24 patients admitted to Brigham and Women's Hospital for suspected stage 1 lung tumors. Informed consent was provided and subjects underwent lung function testing by spirometry and completed a lung health-related questionnaire prior to surgery. Age, height, weight, sex and surgical pathology were obtained from subjects' medical charts. Predicted lung function values (FEV_1_, FVC) were calculated in SAS using the Crapo equations for Caucasians and the Hankinson equations for African-Americans. Diagnosis was confirmed by surgical pathology. A paper describing identification of COPD biomarkers identified by expression profiling from the lung tissue samples has been published previously[17].

### Isolation of PBMC RNA

PBMCs were isolated by using CPT tubes (Becton Dickinson, Franklin Lakes, NJ) according to manufacturer's instructions. Approximately 8 ml whole blood was collected from each subject. Following centrifugation, cells were lysed for RNA isolation. *DNAse-free t*otal RNA was purified using the RNeasy mini kit (Qiagen, Inc, Valencia, CA) according to manufacturer's recommendations. RNA concentrations were determined by Nanodrop ND-1000 (Nanodrop Technologies, Wilmington, DE). RNA quality was assessed on an Agilent 2100 Bioanalyzer; samples with a RNA Integrity Score of > 6.0 were used in this study.

### Microarray Analysis

RNA samples were used for fluorescent target generation (via in-vitro transcription), hybridized, washed, and scanned on U133 plus 2.0 GeneChips (Affymetrix, Santa Clara, CA) according to the manufacturer's instructions. Two independent versions of expression intensities were extracted from raw data files using either RMA or MAS 5.0 algorithms implemented in BioConductor. Gene annotation information was retrieved from the Affymetrix analysis portal (NetAffx http://www.affymetrix.com). Unsupervised clustering with the nonparametric bootstrap [18] was applied to check for undesirable and unanticipated structure or associations among the samples. Reliability of signal intensity measurements was determined using the Detection Call in GCOS, and analysis was restricted to probe sets reliably detected in all cases and/or all controls.

For discrete analysis (cases vs. controls), we applied two independent tests for differential expression on each version of the data set; Bayesian Analysis of Differential Gene Expression (BADGE) [19] and Significance Analysis of Microarrays (SAM) [20]. In SAM False Discovery Rate (FDR) is calculated by computing the number of significant genes for a given threshold for each permutation. The median number of significant genes from these permutations is the median False Discovery Rate. Since genes identified as significant from the permuted data are in fact false positives and as such the median number over many randomizations is a good estimate of false discovery rate. For quantitative analysis, correlation coefficients of signal intensity and lung function (FEV_1 _or FEV_1_/FVC) were calculated. For each probe set, we calculated both the Pearson linear and Spearman rank correlation coefficients for both RMA and MAS5-derived expression intensities using SAS.

### Functional Classification

Functional classification of gene sets was performed using EASE v2.0 [21]. Affymetrix probe set IDs for the selected genes were used as the input list while probe set IDs for all filtered probe set genes served as the background set. Gene ontology categories with an EASE score of less than 0.05 were defined as significantly over-represented. Pathway analysis was performed using Ingenuity Pathway Analysis (IPA) on the set of discrete and quantitative biomarkers to identify canonical pathways that are associated with the peripheral markers determined by expression analysis. Canonical pathways with Fisher Exact test p-values less than 0.05 were identified as significantly dysregulated.

### Molecular Validation

We performed quantitative real-time polymerase chain reaction (qPCR) for the genes identified as discrete and quantitative disease markers using assays from Applied Biosystems (Foster City, CA). qPCR was performed on a Agilent MX3000P (La Jolla, CA) using TaqMan chemistry, as previously described [22]. Gene expression levels were calculated according to the relative expression analysis approach using multiple endogenous controls (*PPIA, GAPDH, ACTB, and HPRT1*). Statistical analysis was performed on individual sample dCt values for each gene using either the parametric Student's t-test or non-parametric Mann-Whitney U-test.

## Results

### Subject Demographics

The studies involved 24 subjects including 12 COPD cases with significant airflow obstruction (defined as FEV_1 _< 70% predicted and FEV_1_/FVC ratio < 0.7) and 12 control subjects with normal lung function (FEV_1 _> 80% predicted and FEV_1_/FVC ratio > 0.7). Cases had an average age of 63 years and average FEV1 of 47% predicted. Controls had an average age of 64 years and an average FEV1 of 99% predicted (Table 1). This population represents a subset of the population for which we have previously reported lung tissue gene expression patterns [17].

**Table 1:** Subject Demographics and Pulmonary Function Shown here are subject demographics and lung function data

Phenotype	Case ID	Array ID	Age	Race	Gender	FEV_1 _%Pred	FVC %Pred	FEV_1_/FVC	Diagnosis
Case 1	2797	987W	70	Caucasian	Male	25.22	37	56	NSC Squamous
Case 2	3589	987I	59	Caucasian	Female	28.12	52	44	NSC Squamous
Case 3	2224	987H	52	Caucasian	Female	31.10	46	52	NSC
Case 4	1576	987U	68	Caucasian	Male	36.29	75	43	Emphysema
Case 5	3660	987K	77	Caucasian	Male	43.34	75	53	NSC Squamous
Case 6	2267	987N	56	Caucasian	Male	46.58	67	56	NSC Squamous
Case 7	3175	987B	75	Caucasian	Male	47.59	94	41	NSC Adeno
Case 8	3043	987A	61	Caucasian	Male	52.12	81	59	NSC Squamous
Case 9	2336	987M	65	African-American	Female	65.66*	99	51	NSC Adeno
Case 10	2195	987E	53	Caucasian	Female	60.36	88	57	NSC Adeno
Case 11	2195	987F	53	Caucasian	Female	60.36	88	57	NSC Adeno
Case 12	3822	987R	64	Caucasian	Male	66.81	94	54	NSC Squamous

*Average*			*62.75*			*45.26*	*74.67*	*51.92*	

Control 1	1769	987X	50	Caucasian	Female	82.69	119	75	NSC Adeno
Control 2	2563	987T	55	Caucasian	Female	87.07	95	76	NSC Adeno
Control 3	3712	987Q	62	Caucasian	Female	87.12	80	86	NSC Squamous
Control 4	2473	987S	77	Caucasian	Female	91.53	81	82	NSC Squamous
Control 5	2254	987J	71	Caucasian	Female	95.00	94	78	NSC Adeno
Control 6	3761	987O	40	Caucasian	Male	95.37	101	82	Carcinoid
Control 7	3143	987C	71	Caucasian	Male	103.28	115	72	NSC Adeno
Control 8	3708	987L	78	Caucasian	Female	104.42	91	85	Metastatic Renal Cell Carcinoma
Control 9	3529	987D	54	Caucasian	Female	105.15	116	75	Unknown
Control 10	3555	987G	55	Caucasian	Male	108.49	118	79	Inflammation
Control 11	1584	987V	68	Caucasian	Female	110.95	117	74	NSC Adeno
Control 12	3769	987P	78	Caucasian	Male	112.01	117	78	NSC-mixed

*Average*			*63.25*			*98.59*	*103.67*	*78.5*	

### Expression Biomarker Discovery

#### Discrete Analysis

**Figure 1:**
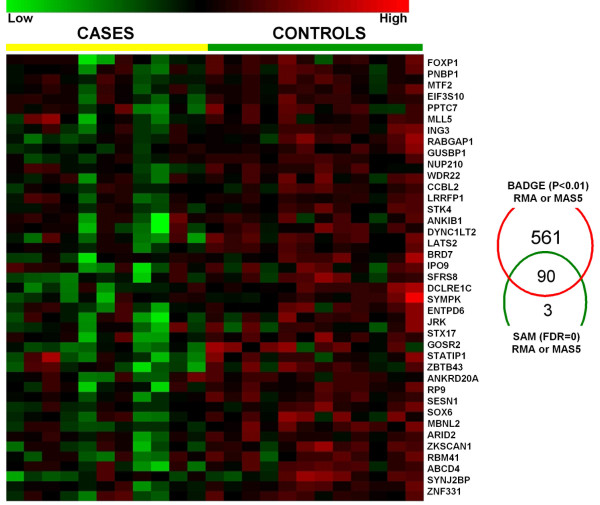
**Discrete biomarkers**. Shown are signal intensity measurements for each of the annotated 45 genes (from 90 probe sets) identified as significantly differentially expressed between cases and controls using both Significance Analysis of Microarrays (SAM) and Bayesian Analysis of Differential Gene Expression (BADGE). Data from individual subjects are in columns and data for individual genes are in rows. Signal intensity data are color-coded such that the intensity of red indicates a relatively high level of expression, while the intensity of green represents a relatively low level of expression (as indicated on scale).

We first extracted signal intensity data using RMA and MAS5, removed data from all probe sets that were not reliably detectable in either all cases or all control samples. We used a stringent set of conditions to identify differential gene expression in this data set, applying multiple significance testing methods (SAM & BADGE). A total of 691 probe sets were significantly different in BADGE analysis at a p-value of 0.01 or less for either RMA or MAS5 versions of data. As our data analysis approach included a combination of multiple tests and normalization approaches, we did not implement any correction on BADGE p-value. SAM analysis identified a total of 93 probe sets that were significantly different at median false discovery rate of 0 (median FDR = 0) in either RMA or MAS5 versions of the data. Ninety (97%) of the probe sets identified in SAM analysis were also identified using BADGE, and represented 47 known genes that we defined as differentially expressed in PBMC from subjects with COPD cases versus controls. Interestingly, all genes identified using these highly stringent criteria were expressed at lower levels in cases as compared to controls (Figure 1). A list of 90 probe sets identified by SAM and BADGE has been provided in Additional File 1 Table S1.

#### Quantitative Analysis

**Figure 2:**
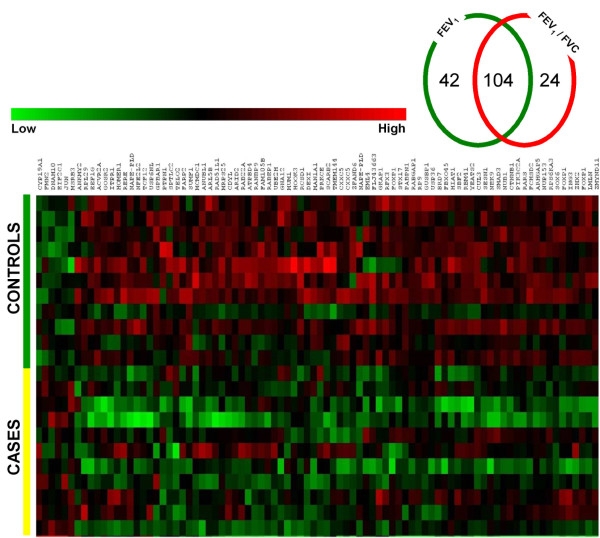
**Quantitative biomarkers**. Shown are signal intensity measurements for 86 annotated genes (among the 104 probe sets) identified as significantly correlated with FEV_1_%predicted and FEV_1_/FVC at P < 0.05. Data from individual subjects are in rows and data for individual genes are in columns. Signal intensity data are color-coded such that the intensity of red indicates a relatively high level of expression, while the intensity of green represents a relatively low level of expression.

**Figure 3:**
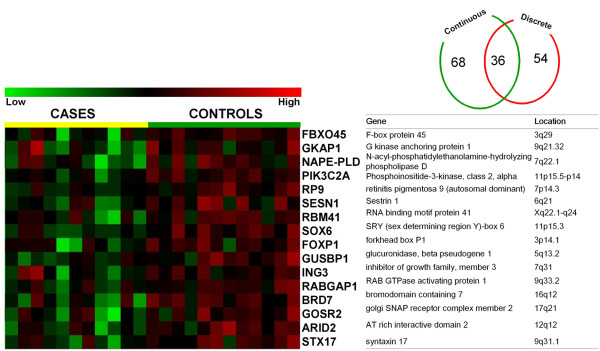
**Biomarkers for both discrete and quantitative phenotypes**. Shown are signal intensity measurements for the 16 genes (among 36 probe sets) identified as significantly different between cases and controls and significantly correlated with both FEV1%predicted and FEV1/FVC. Data from individual subjects are in columns and data for individual genes are in rows. Signal intensity data are color-coded such that the intensity of red indicates a relatively high level of expression, while the intensity of green represents a relatively low level of expression. Complete gene names and chromosomal locations are listed.

We also calculated Pearson's & Spearman rank correlations between expression and FEV_1 _or FEV_1_/FVC for all probe sets. A total of 146 probe sets were significantly correlated with FEV_1 _at p < 0.05 and 9 probe sets were significant at p < 0.01. A total of 128 probe sets were significantly correlated with FEV_1_/FVC at p < 0.05 and 34 probe sets significant at p < 0.01. At a p < 0.05, 104 probe sets (representing 86 known genes) were significantly correlated with both FEV_1 _and FEV_1_/FVC (Figure 2), while at p < 0.01, the overlap was 6 probe sets, representing 2 known genes (*SOX6, LMLN*; both positively correlated with pulmonary function). A list of 104 probe sets significantly correlated with both FEV_1 _and FEV_1_/FVC at p < 0.05 has been provided in Additional File 1 Table S2A and Table S2. There was no overlap among probe sets at p < 0.001. A total of 158 probe sets passed criteria as either discrete (90 probesets) or quantitative (104 probesets at p < 0.05) gene expression markers of COPD. Among these, 36 probe sets representing 16 known genes were significantly different in Case-Control analysis and significantly correlated with both FEV_1 _and FEV_1_/FVC at p < 0.05 (Figure 3).

We assessed whether differences in the distribution of tumor type between Cases and Controls contributed to the identification of these gene expression changes. The tumor types among the 24 subjects included 9 adenocarcinoma and 9 squamous cell carcinoma subjects. We applied differential expression analysis (as described for COPD cases and controls above) comparing all samples classified as adenocarcinoma versus samples classified as squamous cell carcinoma. No probe sets were identified as consistently differentially expressed between tumor types. Further, no probe sets identified as differentially expressed between tumor types in any single analysis were among the COPD biomarker gene set.

### Functional Classification

**Figure 4:**
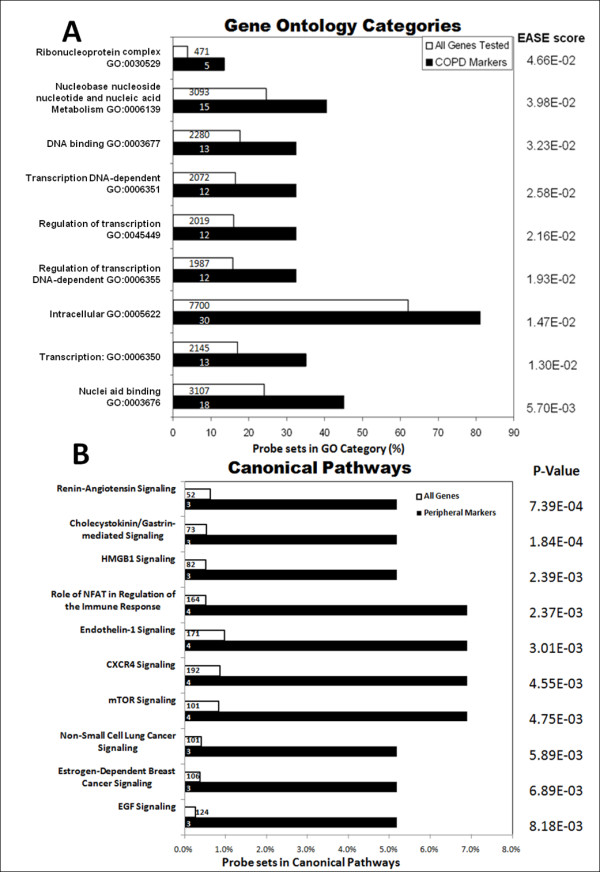
**Functional Classification**. (A) Gene Ontology categories significantly overrepresented in peripheral COPD biomarkers (EASE score < 0.05). Given are GO category name and number, the percentage of genes within the category for COPD markers (black bars) or all genes tested (open bars) and the EASE scores for the category. Number of genes in each category is shown on the bars. (B) Canonical pathways associated with COPD peripheral gene expression markers identified by Ingenuity Pathway Analysis. Shown here are top ten significantly affected canonical pathways, the percentage of genes within the pathway for COPD markers (black bars) or all genes tested (open bars) and the Fisher Exact p-values scores for the pathway. Number of genes in each category is shown on the bars.

In an order to identify biological systems or functions that are associated with discrete or quantitative COPD peripheral gene expression markers, we performed gene ontology assessment using EASE (Figure 4A). We used a set of 158 probe sets that were either significantly different in cases and controls or significantly correlated with lung function and queried for over-represented ontologies using EASE. There was a consistent over-representation of functions relating to transcriptional activity and nucleic acid binding for all sets of COPD biomarkers. A total of 103 probe sets, or 65% of biomarkers tested for ontology (some of the probe sets lacked ontological annotation), were classified in one or more categories related to these functions.

Among all 158 peripheral biomarker genes (both discrete and quantitative), 40 had an annotated molecular function, 37 had an annotated biological process and 30 had an annotated molecular function. Eighteen of 40 genes (45%; p < 0.05) were classified for the molecular function of Nucleic Acid Binding (GO: 0003676). Twelve of 37 genes (32%; p < 0.05) were classified for the biological process of DNA-dependent Transcription (GO: 0006351) and 15 of 37 genes (40%; p < 0.05) were classified for the biological process of nucleoside, nucleoside & nucleotide metabolism (GO::0008150). Among the discrete marker genes (case vs. control), 30 had an annotated molecular function and 27 had an annotated biological process. Thirteen of 30 genes (32%; p < 0.05) were classified for the molecular function of Nucleic Acid Binding (GO: 0003676). Nine of 27 genes (33%; p < 0.05) were classified for the biological process of DNA-dependent Transcription (GO: 0006351) and 11 of 27 genes (40%; p < 0.05) were classified for the biological process of nucleoside, nucleoside & nucleotide metabolism (GO::0008150). Among the quantitative marker genes (correlation), 18 genes had an annotated molecular function and 18 had an annotated biological process. Nine of 18 genes (50%; p < 0.05) were classified for the molecular function of Nucleic Acid Binding (GO: 0003676) and four of 18 genes (22%; p < 0.05) were classified for the molecular function of Transcription factor activity (GO: 0003700). Six of 18 genes (33%; p < 0.05) were classified for the biological process of DNA-dependent Transcription (GO: 0006351) and six of 18 genes (33%; p < 0.05) were classified for the biological process of nucleoside, nucleoside & nucleotide metabolism (GO::0008150). A list of all significantly over-represented Gene Ontology (GO) classes is presented in Additional File 1 Table S3.

Pathway analysis also provided insights to canonical pathways associated with peripheral markers (Figure 4B). Significantly over-represented pathways (p < 0.05) included those associated with cell signaling, inflammatory cell regulation, and cancer; EGF (3 biomarkers out of a total of 171 genes associated with category in IPA), Endothelin-1 (4 biomarkers out of a total of 101 genes) mTOR (4 biomarkers out of a total of 124 genes), CXCR4 (4 biomarkers out of a total of 192 genes), IL-2 (2 biomarkers out of a total of 61 genes), IL-3 (2 biomarkers out of a total of 76 genes), IL-17 (2 biomarkers out of a total of 77 genes), ILK (3 biomarkers out of a total of 191 genes), IL-8 (3 biomarkers out of a total of 193 genes), breast cancer (3 biomarkers out of a total of 106 genes) lung cancer (3 biomarkers out of a total of 101 genes), and glioblastoma (3 biomarkers out of a total of 166 genes). A list of all significant canonical pathways is presented in Additional File 1 Table S4.

### Validation

We performed qPCR-based validation for a subset of genes identified as differentially expressed in COPD subjects using both discrete and quantitative analyses using all samples (n = 24). Validation analysis confirmed significant correlations between microarray-based and qPCR-based expression measures for *GKAP1 *(|r| = 0.25, p < 0.05) and *STX17 *(|r| = 0.36, p < 0.05). However, qPCR did not confirm significant differences in expression for either of these genes between cases and controls.

## Discussion

Even with current advancements in medical technologies, appropriate diagnosis and management of COPD remains a major challenge. Spirometry as a measure of lung function remains the primary objective test for diagnosis of COPD, but spirometry cannot indicate whether airflow obstruction relates to emphysema, airway disease, or both processes. Additional non- or minimally-invasive approaches would be very useful for disease diagnosis and management.

In recent years, studies have attempted to identify gene expression biomarkers for COPD [13-15]. In those studies, genome-wide expression studies have been based on RNA derived from surgically-derived tissue samples. Although gene expression studies of lung tissues may provide useful insights into disease pathogenesis, it is not practical to consider routine COPD diagnosis from a sample that must be obtained through an invasive surgical procedure. Blood samples are less invasive, potentially provide for a larger sample size, and allow repeated sampling to monitor disease progression over time and to study therapeutic response.

Past genome-wide studies on different organ systems have shown that total RNA derived from circulating blood can distinguish between control subjects and patients with various diseases [23-31] including inflammatory (e.g. preeclampsia, rheumatoid arthritis, and chronic pancreatitis) and malignant (chronic lymphocytic leukemia and renal cell carcinoma) diseases [32,33]. One of the earliest demonstrations that gene expression changes in peripheral blood mononucleocytes (PBMCs) were associated with disease was demonstrated on a rat brain model, where acute neural assaults resulted in gene expression changes in PBMCs within 24 hours [34]. In the pulmonary system, Showe et al have used peripheral blood gene expression signatures to identify early-stage lung cancer in at-risk populations [35]. Karimi et al. (2006) showed that *in vitro *exposure of PBMC to cigarette smoke induces production of cytokines in a TLR4-dependent manner [36].

We hypothesized that peripheral blood gene expression patterns could help to improve COPD detection, diagnosis or progression. We assessed genome-wide expression patterns in RNA obtained from PBMCs isolated from a subset of 24 of the study subjects using the Affymetrix U133 Plus 2.0 microarray. Data analysis revealed novel genes that were differentially expressed in PBMCs from COPD patients. The genes we identified have not been previously implicated in COPD disease pathogenesis, and as such are likely to be true markers rather than etiological. We observed two genes, *RP9 *and *NAPE-PLD*, showing decreased expression in both lung tissue and blood of COPD subjects when compared to controls. This suggests that PBMC-derived markers may reflect processes ongoing in diseased tissues. Further, our data serves as a proof-of-principal that peripheral gene expression patterns, defined using minimally invasive samples, can be used to describe COPD.

Genome-wide linkage screens aimed to identify disease-susceptibility genes previously identified three linkage regions (chromosomes 2q33-36, 8pter-22, and 12p13-12) in the Boston Early-Onset COPD cohort [37] which includes the locus for one of the novel genes identified in our study, AT-rich domain 2 (*ARID2*). *ARID2 *is a transcriptional co-activator involved in the regulation of cardiac gene expression [38]. Among other genes displaying changes in expression between cases and controls, some have notable functions. Syntaxin 17 (*STX17*) expression in macrophages is regulated by Colony-stimulating factor 1 (CSF-1), a growth factor controlling the development of macrophages from myeloid progenitor cells [39]. *FOXP1 *is a member of winged-helix/forkhead transcription factors and is important in monocyte differentiation and macrophage function [40]. *SESN1*, a stress inducible sestrin regulated by p53, has been reported to be potent inhibitor of mTOR signaling and regulator of cell growth and proliferation [41].

To our knowledge only two studies have previously explored the value of genome-wide peripheral blood expression assessments in patients with COPD [16,42]; both defining serum protein levels. Hurst et al assessed paired baseline and exacerbation plasma samples from patients with COPD and identified 36 biomarkers using protein arrays [42]. They observed that although systemic biomarkers were not helpful in predicting exacerbation severity, acute-phase response at exacerbation was strongly related to monocyte activity. Pinto-Plata et al used protein array on peripheral blood from COPD patients and identified 30 biomarker clusters [16]. They identified a set of biomarkers correlated with lung function.

One major limitation of the current study is that quantitative real time-PCR (qPCR) validation indicated a potential high false discovery rate. Possible reasons for lack of validation for individual genes include expression levels below sensitivity for the assays used, poor assay specificity, alternative splice forms and inaccuracy of array data. The phenotypic heterogeneity of COPD may also be a cause of limited validation results in the current study. Regardless of the root cause of poor validation, the small size of the current study is a major limitation in the generalization of the results presented. Another limitation of the current study is the diagnosis of lung cancer in most subjects. Recent studies have reported that genetic expression in PBMCs is altered in the context of malignancy [32,43]. Lung cancer and COPD are both typically found in smokers and the diagnosis of lung cancer can serve as an independent predictor for COPD, independent of smoking history. Even though we have previously shown any effects of the tumor on gene expression are not significant in distant, histologically normal lung tissue [17], in the case of PBMCs the presence of tumors may contribute to changes in gene expression. Even though four (PIK3C2A, JUN, FNBP1, ITPR1) of our peripheral biomarkers have been implicated in cancer pathophysiology, none of the PBMC biomarkers were differentially expressed between tumor types (among all subjects, or within cases or controls alone).

In conclusion, we used microarray technology to identify gene expression differences in PBMC obtained from COPD patients and controls. Our data contribute to the understanding of gene expression changes occurring in the blood of patients with obstructive lung disease and provide additional insight into potential mechanisms involved in the disease process. Our data suggest that PBMC may be a source of diagnostic markers. The identification and validation of markers may help to facilitate the development of non-invasive methods for diagnosis, classification of disease subtypes and/or provide a means to define response to therapeutic intervention.

## Competing interests

The authors declare that they have no competing interests.

## Authors' contributions

SB generated data, performed data analyses and wrote the manuscript. ST assisted with sample processing, data generation and analyses. SS assisted with sample processing, data generation and analyses. DLD assisted with data analysis. SDS, EKS and JJR helped design and facilitate the study. RB was responsible for sample collection. TJM helped design the study, provided analytic guidance and wrote the manuscript. All authors read and approved the final manuscript.

## References

[B1] National Institutes of Health National Heart L, and Blood Institute 2009 Chart Book on Cardiovascular, Lung, and Blood Diseases 2009

[B2] Chen JC Mannino DM Worldwide epidemiology of chronic obstructive pulmonary disease Curr Opin Pulm Med 1999 5 2 93 99 10.1097/00063198-199903000-00003 10813258

[B3] Murtagh E Heaney L Gingles J Shepherd R Kee F Patterson C MacMahon J Prevalence of obstructive lung disease in a general population sample: the NICECOPD study Eur J Epidemiol 2005 20 5 443 453 10.1007/s10654-005-1248-8 16080593

[B4] Barnes PJ Chronic obstructive pulmonary disease N Engl J Med 2000 343 4 269 280 10.1056/NEJM200007273430407 10911010

[B5] Hogg JC Chu F Utokaparch S Woods R Elliott WM Buzatu L Cherniack RM Rogers RM Sciurba FC Coxson HO The nature of small-airway obstruction in chronic obstructive pulmonary disease N Engl J Med 2004 350 26 2645 2653 10.1056/NEJMoa032158 15215480

[B6] Barnes TW Afessa B Swanson KL Lim KG The clinical utility of flexible bronchoscopy in the evaluation of chronic cough Chest 2004 126 1 268 272 10.1378/chest.126.1.268 15249470

[B7] Agusti A Soriano JB COPD as a systemic disease Copd 2008 5 2 133 138 10.1080/15412550801941349 18415812

[B8] Janus ED Phillips NT Carrell RW Smoking, lung function, and alpha 1-antitrypsin deficiency Lancet 1985 1 8421 152 154 2857224 10.1016/s0140-6736(85)91916-6

[B9] Larsson C Natural history and life expectancy in severe alpha1-antitrypsin deficiency, Pi Z Acta Med Scand 1978 204 5 345 351 309708 10.1111/j.0954-6820.1978.tb08452.x

[B10] Murray CJ Lopez AD Evidence-based health policy--lessons from the Global Burden of Disease Study Science 1996 274 5288 740 743 10.1126/science.274.5288.740 8966556

[B11] Silverman EK Chapman HA Drazen JM Weiss ST Rosner B Campbell EJ O'Donnell WJ Reilly JJ Ginns L Mentzer S Genetic epidemiology of severe, early-onset chronic obstructive pulmonary disease. Risk to relatives for airflow obstruction and chronic bronchitis Am J Respir Crit Care Med 1998 157 6 Pt 1 1770 1778 9620904 10.1164/ajrccm.157.6.9706014

[B12] McCloskey SC Patel BD Hinchliffe SJ Reid ED Wareham NJ Lomas DA Siblings of patients with severe chronic obstructive pulmonary disease have a significant risk of airflow obstruction Am J Respir Crit Care Med 2001 164 8 Pt 1 1419 1424 11704589 10.1164/ajrccm.164.8.2105002

[B13] Golpon HA Coldren CD Zamora MR Cosgrove GP Moore MD Tuder RM Geraci MW Voelkel NF Emphysema lung tissue gene expression profiling Am J Respir Cell Mol Biol 2004 31 6 595 600 10.1165/rcmb.2004-0008OC 15284076

[B14] Ning W Li CJ Kaminski N Feghali-Bostwick CA Alber SM Di YP Otterbein SL Song R Hayashi S Zhou Z Comprehensive gene expression profiles reveal pathways related to the pathogenesis of chronic obstructive pulmonary disease Proc Natl Acad Sci USA 2004 101 41 14895 14900 10.1073/pnas.0401168101 15469929 PMC522001

[B15] Spira A Beane J Pinto-Plata V Kadar A Liu G Shah V Celli B Brody JS Gene expression profiling of human lung tissue from smokers with severe emphysema Am J Respir Cell Mol Biol 2004 31 6 601 610 10.1165/rcmb.2004-0273OC 15374838

[B16] Pinto-Plata V Toso J Lee K Park D Bilello J Mullerova H De Souza MM Vessey R Celli B Profiling serum biomarkers in patients with COPD: associations with clinical parameters Thorax 2007 62 7 595 601 10.1136/thx.2006.064428 17356059 PMC2117244

[B17] Bhattacharya S Srisuma S Demeo DL Shapiro SD Bueno R Silverman EK Reilly JJ Mariani TJ Molecular biomarkers for quantitative and discrete COPD phenotypes Am J Respir Cell Mol Biol 2009 40 3 359 367 18849563 10.1165/rcmb.2008-0114OC PMC2645534

[B18] Bhattacharya S Long D Lyons-Weiler J Overcoming confounded controls in the analysis of gene expression data from microarray experiments Appl Bioinformatics 2003 2 4 197 208 15130791

[B19] Sebastiani P Yu YH Ramoni MF Bayesian machine learning and its potential applications to the genomic study of oral oncology Adv Dent Res 2003 17 104 108 10.1177/154407370301700124 15126219

[B20] Tusher VG Tibshirani R Chu G Significance analysis of microarrays applied to the ionizing radiation response Proc Natl Acad Sci USA 2001 98 9 5116 5121 10.1073/pnas.091062498 11309499 PMC33173

[B21] Hosack DA Dennis G Jr Sherman BT Lane HC Lempicki RA Identifying biological themes within lists of genes with EASE Genome Biol 2003 4 10 R70 10.1186/gb-2003-4-10-r70 14519205 PMC328459

[B22] Simon DM Arikan MC Srisuma S Bhattacharya S Tsai LW Ingenito EP Gonzalez F Shapiro SD Mariani TJ Epithelial cell PPAR[gamma] contributes to normal lung maturation Faseb J 2006 20 9 1507 1509 10.1096/fj.05-5410fje 16720732

[B23] Cheung W Bluth MJ Johns C Khan S Lin YY Bluth MH Peripheral blood mononuclear cell gene array profiles in patients with overactive bladder Urology 75 4 896 901 10.1016/j.urology.2009.06.021 19775734

[B24] Moore DF Li H Jeffries N Wright V Cooper RA Jr Elkahloun A Gelderman MP Zudaire E Blevins G Yu H Using peripheral blood mononuclear cells to determine a gene expression profile of acute ischemic stroke: a pilot investigation Circulation 2005 111 2 212 221 10.1161/01.CIR.0000152105.79665.C6 15630028

[B25] Achiron A Gurevich M Friedman N Kaminski N Mandel M Blood transcriptional signatures of multiple sclerosis: unique gene expression of disease activity Ann Neurol 2004 55 3 410 417 10.1002/ana.20008 14991819

[B26] Martignoni ME Kunze P Hildebrandt W Kunzli B Berberat P Giese T Kloters O Hammer J Buchler MW Giese NA Role of mononuclear cells and inflammatory cytokines in pancreatic cancer-related cachexia Clin Cancer Res 2005 11 16 5802 5808 10.1158/1078-0432.CCR-05-0185 16115919

[B27] Subrata LS Bizzintino J Mamessier E Bosco A McKenna KL Wikstrom ME Goldblatt J Sly PD Hales BJ Thomas WR Interactions between innate antiviral and atopic immunoinflammatory pathways precipitate and sustain asthma exacerbations in children J Immunol 2009 183 4 2793 2800 10.4049/jimmunol.0900695 19620293

[B28] Sturlan S Sachet M Baumann S Kuznetsova I Spittler A Bergmann M Influenza a virus induces an immediate cytotoxic activity in all major subsets of peripheral blood mononuclear cells PLoS One 2009 4 1 e4122 10.1371/journal.pone.0004122 19125202 PMC2610492

[B29] Wang Y Barbacioru CC Shiffman D Balasubramanian S Iakoubova O Tranquilli M Albornoz G Blake J Mehmet NN Ngadimo D Gene expression signature in peripheral blood detects thoracic aortic aneurysm PLoS One 2007 2 10 e1050 10.1371/journal.pone.0001050 17940614 PMC2002514

[B30] Giusti B Rossi L Lapini I Magi A Pratesi G Lavitrano M Biasi GM Pulli R Pratesi C Abbate R Gene expression profiling of peripheral blood in patients with abdominal aortic aneurysm Eur J Vasc Endovasc Surg 2009 38 1 104 112 10.1016/j.ejvs.2009.01.020 19233690

[B31] Han M Liew CT Zhang HW Chao S Zheng R Yip KT Song ZY Li HM Geng XP Zhu LX Novel blood-based, five-gene biomarker set for the detection of colorectal cancer Clin Cancer Res 2008 14 2 455 460 10.1158/1078-0432.CCR-07-1801 18203981

[B32] Baine MJ Chakraborty S Smith LM Mallya K Sasson AR Brand RE Batra SK Transcriptional profiling of peripheral blood mononuclear cells in pancreatic cancer patients identifies novel genes with potential diagnostic utility PLoS One 6 2 e17014 10.1371/journal.pone.0017014 PMC3037404 21347333

[B33] Rosas IO Richards TJ Konishi K Zhang Y Gibson K Lokshin AE Lindell KO Cisneros J Macdonald SD Pardo A MMP1 and MMP7 as potential peripheral blood biomarkers in idiopathic pulmonary fibrosis PLoS Med 2008 5 4 e93 10.1371/journal.pmed.0050093 18447576 PMC2346504

[B34] Tang Y Lu A Aronow BJ Sharp FR Blood genomic responses differ after stroke, seizures, hypoglycemia, and hypoxia: blood genomic fingerprints of disease Ann Neurol 2001 50 6 699 707 10.1002/ana.10042 11761467

[B35] Showe MK Vachani A Kossenkov AV Yousef M Nichols C Nikonova EV Chang C Kucharczuk J Tran B Wakeam E Gene expression profiles in peripheral blood mononuclear cells can distinguish patients with non-small cell lung cancer from patients with nonmalignant lung disease Cancer Res 2009 69 24 9202 9210 10.1158/0008-5472.CAN-09-1378 19951989 PMC2798582

[B36] Karimi K Sarir H Mortaz E Smit JJ Hosseini H De Kimpe SJ Nijkamp FP Folkerts G Toll-like receptor-4 mediates cigarette smoke-induced cytokine production by human macrophages Respir Res 2006 7 66 10.1186/1465-9921-7-66 16620395 PMC1481582

[B37] Silverman EK Palmer LJ Mosley JD Barth M Senter JM Brown A Drazen JM Kwiatkowski DJ Chapman HA Campbell EJ Genomewide linkage analysis of quantitative spirometric phenotypes in severe early-onset chronic obstructive pulmonary disease Am J Hum Genet 2002 70 5 1229 1239 10.1086/340316 11914989 PMC447597

[B38] Zhang X Azhar G Zhong Y Wei JY Zipzap/p200 is a novel zinc finger protein contributing to cardiac gene regulation Biochem Biophys Res Commun 2006 346 3 794 801 10.1016/j.bbrc.2006.05.211 16782067

[B39] Achuthan A Masendycz P Lopez JA Nguyen T James DE Sweet MJ Hamilton JA Scholz GM Regulation of the endosomal SNARE protein syntaxin 7 by colony-stimulating factor 1 in macrophages Mol Cell Biol 2008 28 20 6149 6159 10.1128/MCB.00220-08 18710945 PMC2577439

[B40] Shi C Sakuma M Mooroka T Liscoe A Gao H Croce KJ Sharma A Kaplan D Greaves DR Wang Y Down-regulation of the forkhead transcription factor Foxp1 is required for monocyte differentiation and macrophage function Blood 2008 112 12 4699 4711 10.1182/blood-2008-01-137018 18799727 PMC2597137

[B41] Budanov AV Karin M p53 target genes sestrin1 and sestrin2 connect genotoxic stress and mTOR signaling Cell 2008 134 3 451 460 10.1016/j.cell.2008.06.028 18692468 PMC2758522

[B42] Hurst JR Donaldson GC Perera WR Wilkinson TM Bilello JA Hagan GW Vessey RS Wedzicha JA Use of plasma biomarkers at exacerbation of chronic obstructive pulmonary disease Am J Respir Crit Care Med 2006 174 8 867 874 10.1164/rccm.200604-506OC 16799074

[B43] Burczynski ME Twine NC Dukart G Marshall B Hidalgo M Stadler WM Logan T Dutcher J Hudes G Trepicchio WL Transcriptional profiles in peripheral blood mononuclear cells prognostic of clinical outcomes in patients with advanced renal cell carcinoma Clin Cancer Res 2005 11 3 1181 1189 15709187

